# Psychotic Manifestations in Huntington’s Disease: A Systematic Review of Clinical Presentation, Treatment Approaches, and Outcomes

**DOI:** 10.1192/bjo.2025.10249

**Published:** 2025-06-20

**Authors:** Aliu Yakubu, Moses Effiong, Olorungbami Anifalaje, Oluwakemi Olalude, Francess Adeyemi

**Affiliations:** 1 University Hospital Wishaw, Wishaw, United Kingdom; 2 Glasgow Caledonian University, Glasgow, United Kingdom; 3 NHS Dumfries and Galloway, Dumfries, United Kingdom; 4 Lagos State University Teaching Hospital, Ikeja, Nigeria; 5 Afe Babalola University, Ado Ekiti, Nigeria

## Abstract

**Aims:** Huntington’s disease (HD) is a progressive neurodegenerative disorder characterized by motor dysfunction, cognitive decline, and psychiatric symptoms. Among the psychiatric manifestations, psychotic symptoms such as delusions and hallucinations remain underreported and poorly understood. This systematic review aims to analyse the prevalence, clinical presentation, assessment tools, and treatment approaches for psychosis in HD patients.

**Methods:** A comprehensive literature search (PubMed, Embase, PsycINFO, and Google Scholar) was conducted using multiple electronic databases to identify studies reporting psychotic symptoms in HD patients. Inclusion criteria involved case reports that provided detailed psychiatric presentations, genetic findings, and treatment interventions. Data extracted included patient demographics, genetic CAG repeat counts, psychiatric symptoms, assessment tools, pharmacological and non-pharmacological interventions, side effects, and patient outcomes.

**Results:** A total of 53 case reports with 60 cases were included. The mean age of psychiatric symptom onset was 42.58 years, and the mean age at HD diagnosis was 38.09 years. The mean CAG repeat count was 44.71, and the mean age of onset of motor symptoms was 41.25 years. The review identified multiple cases of HD patients presenting with psychotic symptoms, including persecutory and grandiose delusions, auditory and visual hallucinations, and paranoia. Electroconvulsive therapy (ECT) was used in 20% of cases. The most frequently used antipsychotics were risperidone (26.7%), olanzapine (23.3%), clozapine (16.7%), aripiprazole (16.7%), and haloperidol (13.3%). A family history of HD was present in 73.3% of patients. In 36.6% of cases, side effects were reported, including extrapyramidal side effects, orthostatic hypotension, neutropenia, and increased sedation. In 93.3% of cases, symptomatic improvement was reported, though some patients exhibited persistent cognitive dysfunction and residual psychotic features.

**Conclusion:** Psychotic symptoms in HD are a significant but understudied phenomenon, frequently complicating disease management. The findings highlight the need for standardized assessment protocols and tailored treatment approaches to mitigate psychiatric distress while balancing cognitive and motor function preservation. Future research should focus on longitudinal studies to better understand the trajectory of psychosis in HD and optimize treatment strategies.

